# CD38-Induced Apoptosis and Mitochondrial Damage is Restored by Nicotinamide in Prostate Cancer

**DOI:** 10.3389/fmolb.2022.890402

**Published:** 2022-05-23

**Authors:** Mayuko Kanayama, Jun Luo

**Affiliations:** Department of Urology, James Buchanan Brady Urological Institute, School of Medicine, Johns Hopkins University, Baltimore, MD, United States

**Keywords:** prostate cancer, NAD, CD38, mitochodria, nicotinamide (NAM)

## Abstract

Nicotinamide adenine dinucleotide (NAD^+^) is an essential molecule for living organisms. CD38 is a key NAD^+^-dependent enzyme which breaks down NAD^+^ to cyclic ADP-ribose (ADPR) and nicotinamide (NAM, vitamin B3), and NAM can be recycled to synthesize NAD^+^. CD38 expression is consistently silenced by methylation in prostate cancer and progressively downregulated in advanced castration-resistant prostate cancer, suggesting a connection between NAD^+^ and prostate carcinogenesis as well as prostate cancer progression. However, the functional interplay between NAD^+^, CD38, and NAM remains largely uncharacterized in prostate cancer cells. In this study, we generated stable LNCaP95 cell clones expressing varying levels of CD38 upon induction by doxycycline. We demonstrate that CD38 overexpression resulted in growth suppression and apoptosis accompanied by cleavage of poly (ADP-ribose) polymerase 1 (PARP1). CD38 overexpression also dramatically reduced intracellular NAD^+^ levels and decreased mitochondrial respiration as measured by oxygen consumption rate. We further show that some but not all of these CD38-induced phenotypes could be rescued by exogenous NAM. Treatment of cells with NAM rescued CD38-induced apoptosis and mitochondrial stress but did not restore intracellular NAD^+^ levels. We also found that NAM demonstrated biphasic effect on mitochondria function, a finding that can be explained by the dual role of NAM as both a precursor of NAD^+^ and also as a suppressor of a number of NAD^+^-dependent enzymes. Collectively, these findings provide additional insight supporting the functional relevance of CD38 loss in prostate cancer by linking cell-autonomous regulation of mitochondrial function and prostate cancer.

## Introduction

Nicotinamide adenine dinucleotide (NAD^+^) is an essential cofactor in cellular metabolic processes (Demarest et al., 2019). NAD^+^ levels demonstrate age-related decline which is in turn linked to decline of mitochondrial and stem cell function (Zhang et al., 2016). In mitochondria, NADH (NAD^+^ bound by hydrogen) generated in TCA cycle from NAD^+^ is necessary for oxidative phosphorylation to produce ATP (Martínez-Reyes and Chandel, 2020). NAD^+^ is also a substrate for NAD^+^-dependent enzymes that can be grouped in three main categories: poly (ADP-ribose) polymerases (PARPs), CD38, and sirtuins (Demarest et al., 2019). These NAD^+^-dependent enzymes results in breakdown of NAD^+^ to cyclic ADP-ribose (ADPR) and nicotinamide (NAM, aka vitamin B3), and NAM is recycled into NAD^+^ by the salvage NAD^+^ synthesis pathway (Chiarugi et al., 2012). Other than the salvage pathway, NAD^+^ can be synthesized through the *de novo* NAD^+^ synthesis pathway. Through the *de novo* NAD^+^ synthesis pathway, NAD^+^ is synthesized in liver from tryptophan and liver also releases NAM into the circulation. Other tissues, by contrast, rely on NAM released by liver as the NAD^+^ precursor to generate NAD^+^ by the salvage pathway (Liu et al., 2018). For most tissues including prostate, NAM is considered to be the most common source of NAD^+^ in normal physiological conditions.

Among the three categories of NAD^+^-dependent enzymes, CD38 is of particular relevance to prostate cancer because its expression is downregulated in prostate cancer. In one of our earlier expression microarray data set comparing prostate cancer tissues with paired adjacent normal prostate tissues (Dunn et al., 2006), CD38 was among one of the most consistently downregulated genes in prostate cancer. This observation, however, was not further followed up and investigated within our research group. Findings from recent studies provided important insights in relation to the role of CD38 in prostate cancer (Liu et al., 2016; Chmielewski et al., 2018; Mottahedeh et al., 2018; Guo et al., 2021). These recent findings suggest that silencing CD38 (through methylation) may confer survival advantage to tumor-initiating progenitor cells or proliferative cancer cells by increasing NAD^+^ availability and mitochondrial function (Chiarugi et al., 2012; Camacho-Pereira et al., 2016; Liu et al., 2016; Chmielewski et al., 2018; Mottahedeh et al., 2018). These studies also suggest CD38 expression at the bulk tissue level was progressively lower in more advanced castration-resistant prostate cancers. CD38 expression however remain detectable in bulk prostate cancer tissues mainly due to its expression in cell types other than the prostate cancer cells. For example, within the tumor microenvironment, expression of CD38 in general is limited to tumor-infiltrating immune cells (TIICs) (Guo et al., 2021). In this setting, CD38 expression is associated with prostate cancer progression, presumably due to its extracellular enzymatic activity leading to the synthesis of immunosuppressive adenosine (Horenstein et al., 2013; Guo et al., 2021). The role of CD38 in TIICs is consistent with elevated CD38 in a number of hematological cancers and the clinical effectiveness of anti-CD38 monoclonal antibodies (e.g., daratumumab) in treating multiple myeloma (Van De Donk and Usmani, 2018). On the basis of these studies, it appears that CD38 may have different functions and relevance to disease progression depending on the cell type it is expressed in.

Functional studies focusing on CD38 in prostate cancer have relied on prostate cancer cell lines. Prostate cancer cell lines are negative for CD38 and have been used to investigate the function of CD38 following gene overexpression (Chmielewski et al., 2018; Mottahedeh et al., 2018). These two studies reported contradictory findings with regard to the regulation of intracellular NAD^+^ by CD38 (Chmielewski et al., 2018; Mottahedeh et al., 2018). Mottahedeh et al. (2018) reported that CD38 reduced extracellular NAD^+^ levels but did not affect intracellular NAD^+^ levels, whereas Chmielewski et al. (2018) showed that CD38 overexpression resulted in reduced intracellular NAD^+^. Intracellular functional role of CD38 would imply a cell-autonomous mechanism of function while an extracellular manifestation of the functional readout would suggest influence on the tumor microenvironment. Therefore, additional studies on this topic are warranted. In addition, the role of NAM in regulating CD38-induced phenotypic changes remains unknown. In this study, we evaluated the functional impact of CD38 overexpression on cellular viability, intracellular NAD^+^ level, and mitochondrial respiration following induced expression of CD38 in stable prostate cancer cell clones derived from the CD38 negative, castration-resistant LNCaP95 cell line, and tested whether NAM rescues CD38-induced phenotypes by restoring NAD^+^ level. Study findings support a role of CD38 in regulating intracellular NAD^+^. Although NAM failed to restore NAD^+^, it did rescue the functional phenotypes mediated by CD38 expression and depletion of NAD^+^ which may be explained by active usage and breakdown of NAD^+^ derived from NAM by overexpressed CD38. We also document a biphasic function of NAM in regulation of mitochondria function and discussed implications of the findings.

## Materials and Methods

### Cell Lines and Treatments

LNCaP95 is an androgen-independent prostate cancer cell line derived from the parental LNCaP (Hu et al., 2012). LNCaP95 was cultured in RPMI 1640 media without phenol red (Thermo Fisher Scientific) supplemented with 10% charcoal-stripped FBS (Invitrogen). Nicotinamide (NAM) was obtained from Sigma and prepared as a 1 M NAM solution used to treat the cells with final concentrations of 5, 10, and 20 mM.

### CD38-Inducible LNCaP95 Stable Lines

Tet-ON 3G bidirectional inducible expression system (Clontech, #631337) was used to generate CD38-inducible stable lines. Briefly, cDNA open reading frame (ORF) of *CD38* gene was purchased from Dharmacon (Clone ID #4309086). CD38 ORF was excised from a plasmid with BglI and XhoI and inserted into the EcoRV-digested pTRE3G-BI vector, followed by EGFP insertion into the multi-cloning site of pTRE3G-BI vector. CD38-inducible stable lines were established in accordance with the manufacturer’s protocol. First, clones stably expressing rtTA were isolated and a clone expressing the highest level of rtTA determined by qRT-PCR was subjected to the second round of transfection with the pTRE3G-BI vector carrying CD38 and EGFP. Stable clones that express CD38 and EGFP upon doxycycline (Dox) (Sigma) treatment were selected. To induce gene expression, 100 ng/ml Dox was used for all experiments with the exception of experiments relating to oxygen consumption rate measurement in which 20 ng/ml Dox was selected because oxygen consumption rate in control cells were not affected at this concentration.

### Cellular Viability Assays

For crystal violet staining, 2×10^5^ cells were seeded into 6-well plates and cultured with or without 100 ng/ml Dox for 4 days. Then, cells were stained with 1 × PBS crystal violet staining solution containing 0.05% crystal violet, 1% formaldehyde and 1% methanol. For IncuCyte analysis, 1×10^4^ cells were seeded into 96-well plates coated with poly-l-lysine 2 days before the analysis. On the day of analysis, fresh culture medium containing 0.5 μM caspase-3/7 red dye was added to evaluate apoptosis (Sartorius, #4704) in the presence or absence of 100 ng/ml Dox, together with indicated concentrations of NAM (in NAM rescue experiments only). Cells were monitored by IncuCyte live cell imaging system by scanning every 2 h for 5 days. Data was analyzed by the InCucyte analysis software (version 2020B). Integrated red fluorescence intensity per well was normalized by confluency.

### Intracellular NAD^+^ and NADH Measurements

To measure intracellular NAD^+^ and NADH levels, cells were seeded onto 10 cm dishes and treated with or without 100 ng/ml Dox and the indicated concentrations of NAM for 48 h (in NAM rescue experiments only). The live cell numbers were counted prior to the assay with trypan blue staining using Countess automated cell counter (Invitrogen), and 2.5 × 10^6^ of live cells were subjected to NAD^+^ and NADH measurements by NAD^+^/NADH assay kit (Abcam #ab65348). Absorbance was measured at 450 nm after 40-min incubation.

### Measurements of Oxygen Consumption Rate

Seahorse XF Cell Mito stress test kit (Agilent #103015-100) was used to measure oxygen consumption rate (OCR). Cells were seeded onto poly-l-lysine-coated XF96 microplates (Agilent) at 2.5 × 10^4^ cells per well and treated with or without NAM and 20 ng/ml Dox for 48 h. Before the assay, culture media was changed to XF medium base medium supplemented with 2 mM l-glutamine, 1 mM pyruvate and 10 mM glucose, followed by 1-h incubation in non-CO_2_ incubator. For injection compounds, the final concentration of 2 μM oligomycin, 250 nM FCCP and 0.5 μM rotenone/antimycin was used. OCR was measured by a Seahorse XF96 analyzer (Agilent). All OCR values were normalized with cell numbers quantified by CyQUANT (Invitrogen #C7026). A total of 12 OCR measurements were made during the assay. For pairwise and multiple comparisons, OCR values at the first measurement point and eighth measurement point were used for basal OCR and maximal OCR, respectively.

### Western Blot

Cells were treated with or without 100 ng/ml Dox and indicated concentrations of NAM for 48 h. Cells were washed with PBS and lysed in buffer containing 1 × passive lysis buffer (Promega) and protease inhibitor (Roche). Protein concentrations were determined using BCA assay (Thermo Fisher Scientific) and 20 μg of protein was separated on 4-15% SDS-PAGE gel (Bio-Rad). Proteins were transferred to PVDF membranes followed by incubation with the following primary antibodies: CD38 (Cell Signaling Technology #51000), PARP1 (Cell Signaling Technology #9532) and β-actin (Sigma #A2228). Signals were detected by enhanced chemiluminescence (Thermo Fisher Scientific).

### qRT-PCR Analysis

Cells were treated with indicated concentrations of Dox and NAM for 48 h and lysed in TRIzol reagent (Invitorogen). Total RNA was extracted using RNeasy mini kit (QIAGEN), followed by cDNA synthesis with SuperScript IV first-strand synthesis system (Invitrogen). iQ SYBR Green Supermix (BioRad) was used for qRT-PCR with the following primer sets: CD38 forward: 5′-GTA​GAC​TGC​CAA​AGT​GTA​TGG​GAT​G-3’; CD38 reverse: 5′-GGG​CCA​GAT​CTT​TTA​TTC​TGC​TCC-3’; GAPDH forward: 5′-AGC​ACC​AGG​TGG​TCT​CCT​C-3’; GAPDH reverse: 5′-CCC​TGT​TGC​TGT​AGC​CAA​ATT​C-3’. Changes in gene expression were compared by the comparative Ct method. LN95-CD38^low^ treated with 100 ng/ml Dox (without NAM) was used as a reference sample.

### Statistical Analysis

All the statistical analyses were performed using GraphPad Prism ver. 9. Comparisons were made with unpaired two-tailed *t* tests. For multiple comparisons, groups were first compared with one-way ANOVA. If there were statistical differences among groups as determined by ANOVA (*P* < 0.05), Turkey’s multiple comparison tests were performed for pairwise comparison.

## Results

### Generation of CD38-Inducible Stable Lines

**FIGURE 1:**
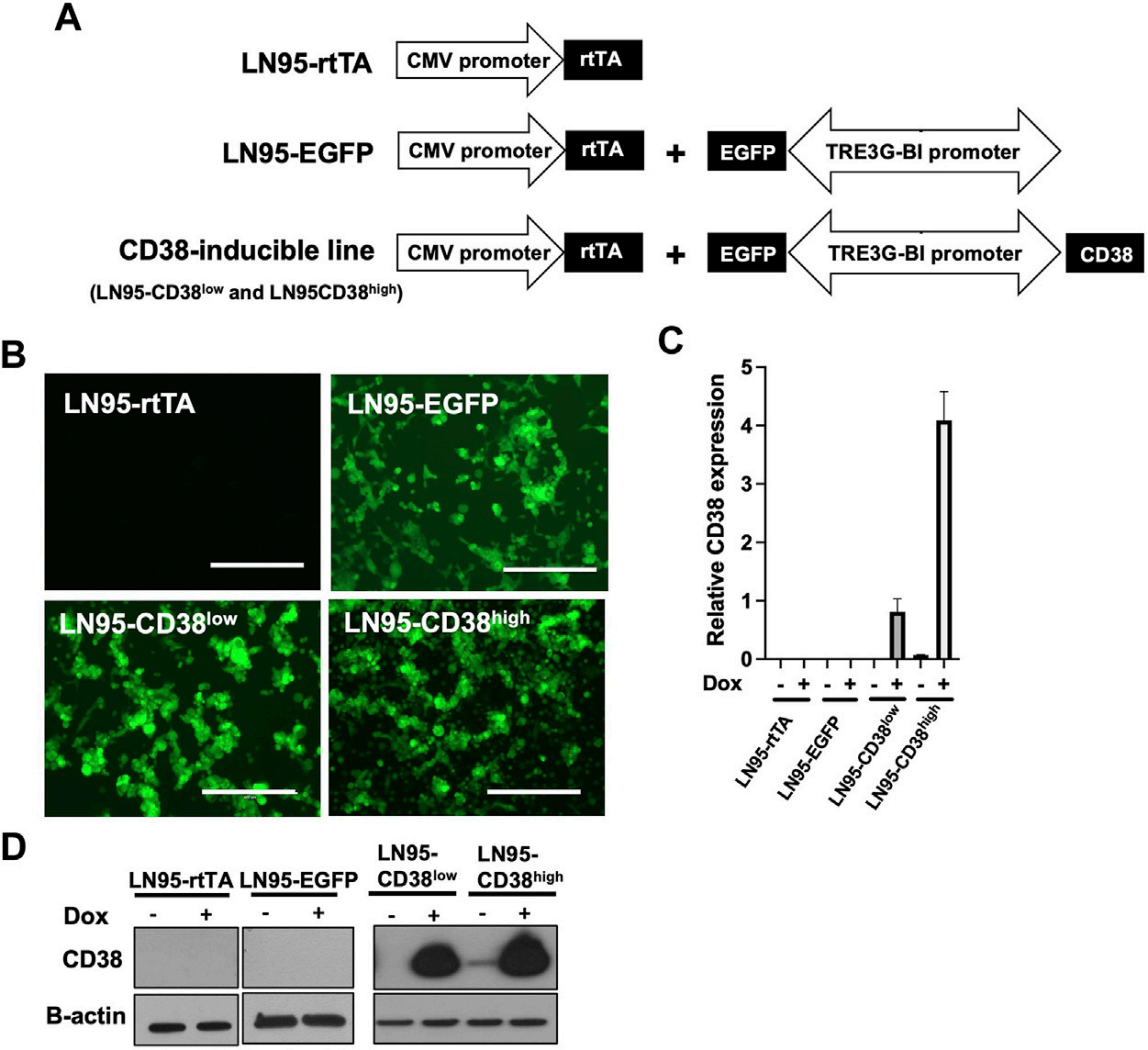
Generation of CD38-inducible stable lines. **(A)** A schematic presentation of CD38-inducible stable lines and controls. **(B)** EGFP emission observed by fluorescence microscope following 100 ng/ml Dox for 48 h. Scale bars: 400 μm. **(C)** CD38 expression level determined by qRT-PCR following 100 ng/ml Dox for 48 h. Mean ± SD values from four replicates are shown. **(D)** CD38 induction confirmed by Western blot analysis after 48-h incubation with or without 100 ng/ml Dox.

To investigate the role of CD38, we utilized the Tet-ON system to generate Dox-inducible stable lines using the CD38 negative castration-resistant LNCaP95 prostate cancer cell line. In the process of generating the stable lines, two clones expressing different levels of Dox-inducible CD38 (hereafter called LN95-CD38^low^ and LN95-CD38^high^) were established, along with a clone stably expressing rtTA without EGFP or CD38 (hereafter called LN95-rtTA), as well as a clone stably expressing rtTA and inducible EGFP without CD38 (hereafter called LN95-EGFP) (Figure 1A). EGFP emission of Dox-treated cells detected by fluorescence microscope confirmed the homogeneous expression of the transgenes (Figure 1B). Further confirmation by qRT-PCR showed approximately 4-fold higher CD38 expression upon Dox treatment in the LN95-CD38^high^ clone than the LN95-CD38^low^ clone (Figure 1C). CD38 protein levels in the 4 clones (LN95-CD38^low^ and LN95-CD38^high^, LN95-rtTA, LN95-EGFP) were further confirmed by Western blot analysis (Figure 1D).

### CD38 Induces Apoptosis

**FIGURE 2:**
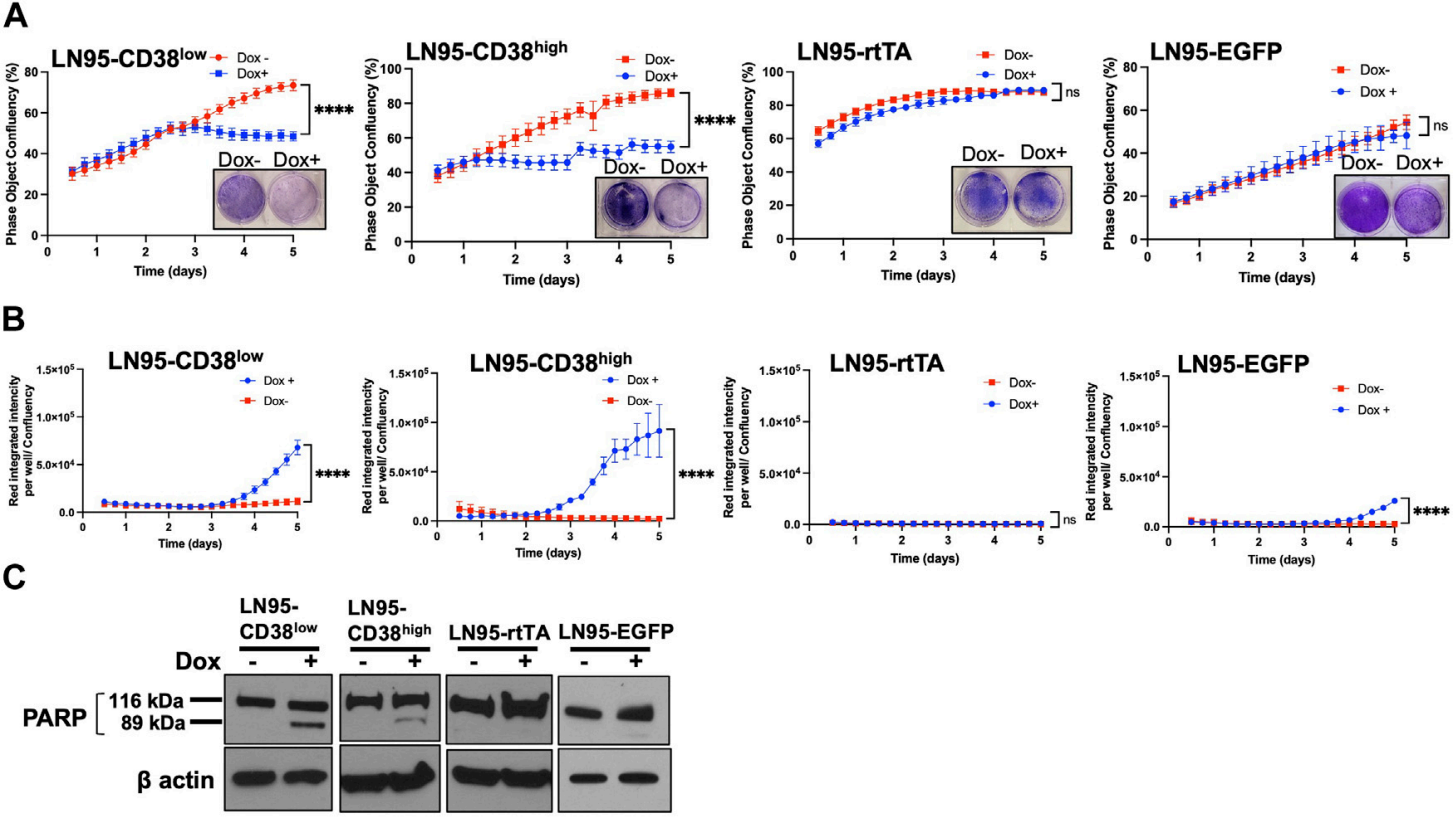
Apoptosis induced by CD38. **(A)** Cell growth with or without 100 ng/ml Dox measured by IncuCyte and crystal violet staining. Confluency was measured by phase-contrast images every 2 hrs for 5 days. Mean ± SD values from four replicates are shown. Crystal violet staining was performed on Day 4. ns: not significant, ****: *p <* 0.0001. **(B)** Apoptosis assay by IncuCyte. Mean ± SD values from four replicates are shown. ns: not significant, ****: *p <* 0.0001. **(C)** Western blot analysis of PARP1 cleavage on Day 2.

The effect of CD38 on cell viability and apoptosis was determined by crystal violet staining and IncuCyte analysis. Both crystal violet staining and real-time monitoring of cell confluency by IncuCyte detected cell growth suppression following Dox treatment (i.e., inducible CD38 expression) in both LN95-CD38^high^ and LN95-CD38^low^ clones but not in LN95-rtTA and LN95-EGFP clones (Figure 2A). This finding is consistent with the role of CD38 in suppressing cell growth (Figure 2A). Next, cells undergoing caspase-3/7-mediated apoptosis were quantified by IncuCyte. CD38 significantly induced apoptosis in both LN95-CD38^low^ and LN95-CD38^high^ clones (Figure 2B). Although increased apoptosis was observed in LN95-EGFP, this is expected because EGFP overexpression was known to induce apoptosis and oxidative stress and this observation is consistent with previously reported toxicity of EGFP (Ansari et al., 2016; Ganini et al., 2017). Nevertheless, the apoptosis rate was much greater in the CD38-expressing clones, again in line with the expectation that the observed phenotypes are mediated by CD38 (as opposed to EGFP). PARP1 is known to be cleaved by caspases during apoptosis (Germain et al., 1999). Consistent with the increased apoptosis mediated by CD38, cleaved bands of PARP1 were detected in LN95-CD38^low^ and LN95-CD38^high^ clones but not in the LN95-rtTA clone (Figure 2C). A weak signal of cleaved band in LN95-EGFP is consistent with toxicity of EGFP (Figure 2C).

### CD38 Depletes Intracellular NAD^+^ and Moderately Reduces Mitochondrial Respiration

**FIGURE 3:**
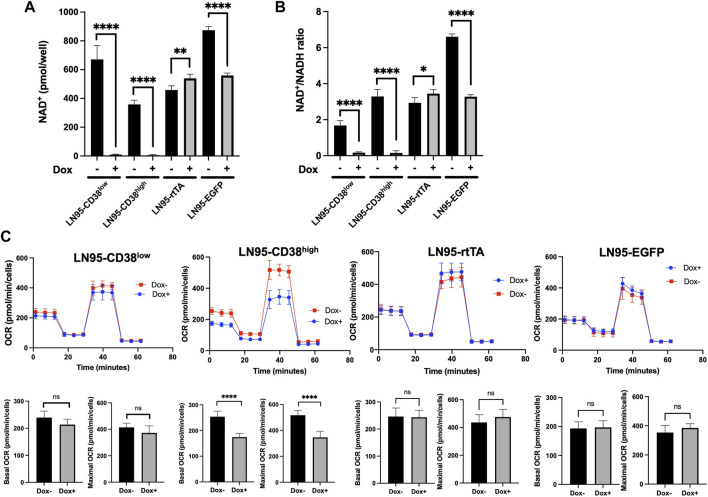
The impact of CD38 expression on intracellular NAD^+^ and mitochondrial respiration. **(A)** Intracellular NAD^+^ following 100 ng/ml Dox for 48 h *: *p* < 0.05, **: *p* < 0.01, ****: *p <* 0.0001. **(B)** NAD^+^/NADH ratios following 100 ng/ml Dox for 48 h. Mean ± SD values from four replicates are shown. *: *p* < 0.05, **: *p* < 0.01, ****: *p <* 0.0001. **(C)** OCR measured by mitochondrial stress test following 20 ng/ml Dox for 48 h. Basal OCR (measurement point 1) and maximal OCR (measurement point 8) were compared. Mean ± SD values from five replicates are shown. ns: not significant, ****: *p <* 0.0001.

Because CD38 breaks down NAD^+^, we next evaluated the intracellular NAD^+^ levels following Dox treatment in the 4 clones. CD38 depleted intracellular NAD^+^ to almost undetectable levels in both LN95-CD38^low^ and LN95-CD38^high^ clones (Figure 3A). The cellular NAD^+^/NADH ratios also significantly decreased upon CD38 expression in both LN95-CD38^low^ and LN95-CD38^high^ clones (Figure 3B). Given that NAD^+^/NADH ratios are reported to be 2-4 (Gaikwad et al., 2001), low NAD^+^/NADH ratios induced by CD38 presumably reflects impaired cellular viability. In the control clones, we did not observe a decrease in either the NAD^+^ level or the NAD^+^/NADH ratios in the LN95-rtTA clone. Although a decrease in the NAD^+^ level and NAD^+^/NADH ratios was detected in the Dox-treated LN95-EGFP clone (Figures 3A,B), similar to the slightly increased apoptosis rate detected in this clone (Figure 2B), the extent of decrease was much smaller than the dramatic decrease observed in the LN95-CD38^low^ and LN95-CD38^high^ clones.

Since NAD^+^ is an essential cofactor for mitochondrial respiration (Stein and Imai, 2012; Martínez-Reyes and Chandel, 2020), mitochondrial stress test was performed to determine the effects of NAD^+^ depletion induced by CD38 on oxygen consumption rate (OCR). Although we used 100 ng/ml Dox for CD38 induction, lower Dox doses were effective in inducing CD38 (data not shown). To better control the OCR measurements that are sensitive to drug treatments, we selected 20 ng/ml as the dose for Dox treatment for the purpose of evaluating the effect of CD38 on OCR, because OCR was not affected in the two control cell clones in our dose-finding experiments (data not shown). As shown in Figure 3C, treatment with this lower Dox doses did not decrease OCR in the LN95-rtTA and LN95-EGFP clones (OCR was in fact slightly increased). In contrast, a decrease in both basal and maximum OCR was detected in both LN95-CD38^low^ (not statistically significant) and LN95-CD38^high^ (statistically significant) clones. Although the results are largely consistent with a role of CD38 in regulating mitochondrial function, the extent of mitochondria impairment following depletion of NAD^+^ was lower than expected, suggesting that cells may be capable of maintaining mitochondrial function for a certain duration under unusually low NAD^+^ levels (as observed in LN95-CD38^low^ clone).

### NAM Rescued CD38-Induced Apoptosis but Failed to Restore Intracellular NAD^+^

**FIGURE 4:**
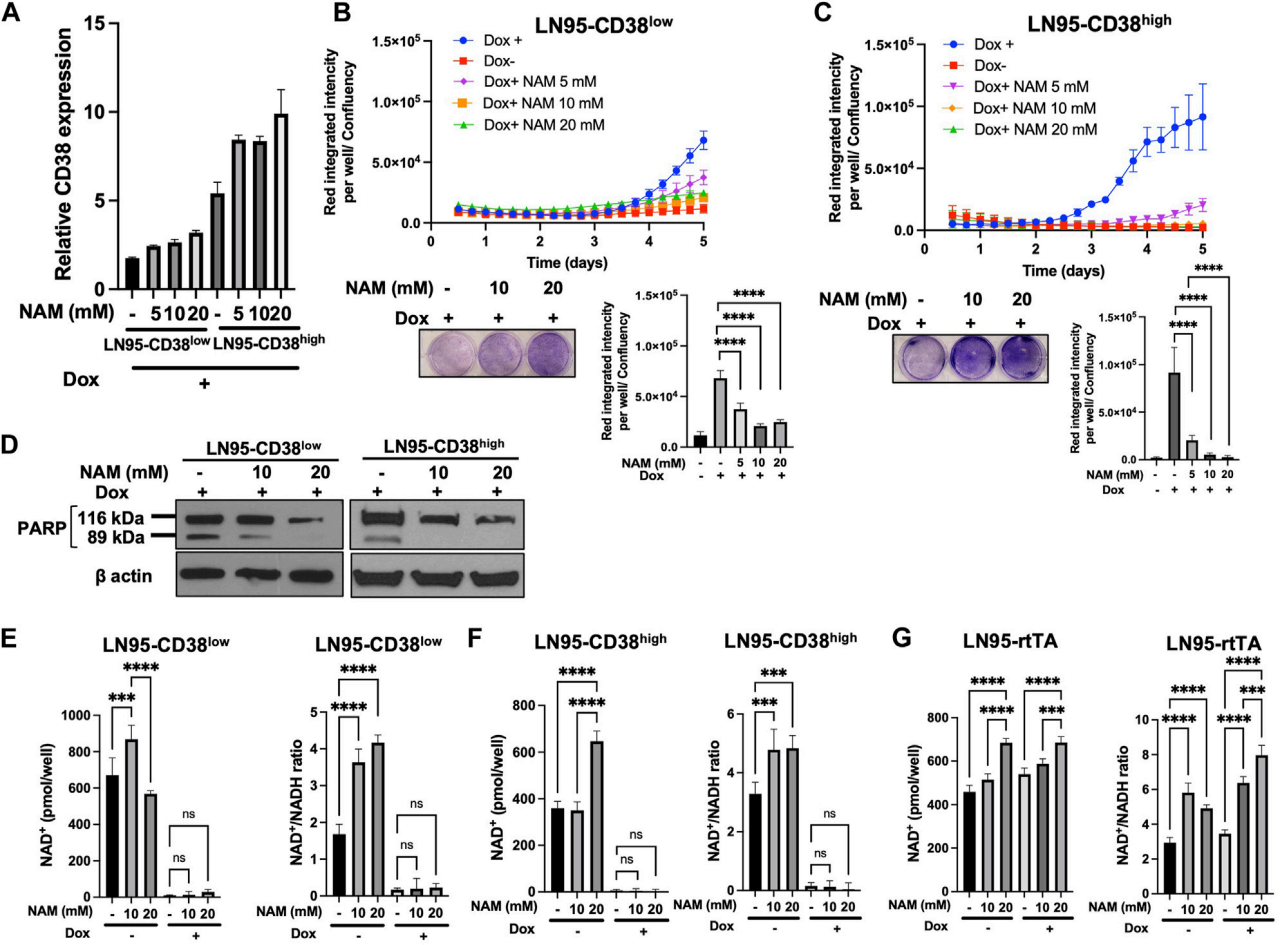
Rescue of CD38-induced apoptosis by NAM. **(A)** CD38 expression level determined by qRT-PCR following 100 ng/ml Dox and NAM for 48 h **(B,C)** Apoptosis assay by IncuCyte and crystal violet staining in LN95-CD38^low^ **(B)** and LN95-CD38^high^ **(C)**. Mean ± SD values from four replicates are shown. *p* values for Turkey’s multiple comparison tests are indicated in each figure. Crystal violet staining was performed on Day 4. ****: *p <* 0.0001. **(D)** Western blots showing inhibition of CD38-induced PARP1 cleavage by NAM. **(E,F,G)** Measurements of NAD^+^ and NAD^+^/NADH ratios in LN95-CD38^low^ **(E)**, LN95-CD38^high^ **(F)**, and LN95-rtTA **(G)** following NAM and 100 ng/ml Dox for 48 h. Mean ± SD values from four replicates are shown. *p* values for Turkey’s multiple comparison tests are indicated in each figure. ns: not significant. ****: *p <* 0.0001, ***: *p <* 0.001.

Given that NAM is a NAD^+^ precursor (Chiarugi et al., 2012), we tested whether NAM supplementation could rescue CD38-induced apoptosis and restore NAD^+^ level. First, we confirmed that NAM treatments do not result in decreased CD38 expression by qRT-PCR (Figure 4A). Cellular viability was evaluated with or without NAM by crystal violet staining and IncuCyte. In both LN95-CD38^low^ and LN95-CD38^high^ clones, we observed significantly reduced apoptosis rate at all the NAM concentrations (5 mM, 10 mM, 20 mM) tested (Figures 4B,C). In line with this finding, NAM inhibited apoptosis-associated PARP1 cleavage detected following Dox-induced CD38 expression (Figure 4D). Next, intracellular NAD^+^ was measured with or without NAM supplementation. Surprisingly, NAM did not increase intracellular NAD^+^ or NAD^+^/NADH ratios in Dox-treated cells (Figures 4E,F), although there was a trend for a moderate increase in NAD^+^ and NAD^+^/NADH ratios after NAM treatments in Dox-untreated cells and the control LN95-rtTA cells (Figure 4G). These findings suggest that restoration of steady rate NAD^+^ may not be necessary to restore cell growth in cells overexpressing CD38 (see Discussion).

### 10 mM NAM Restores Mitochondrial Respiration

**FIGURE 5:**
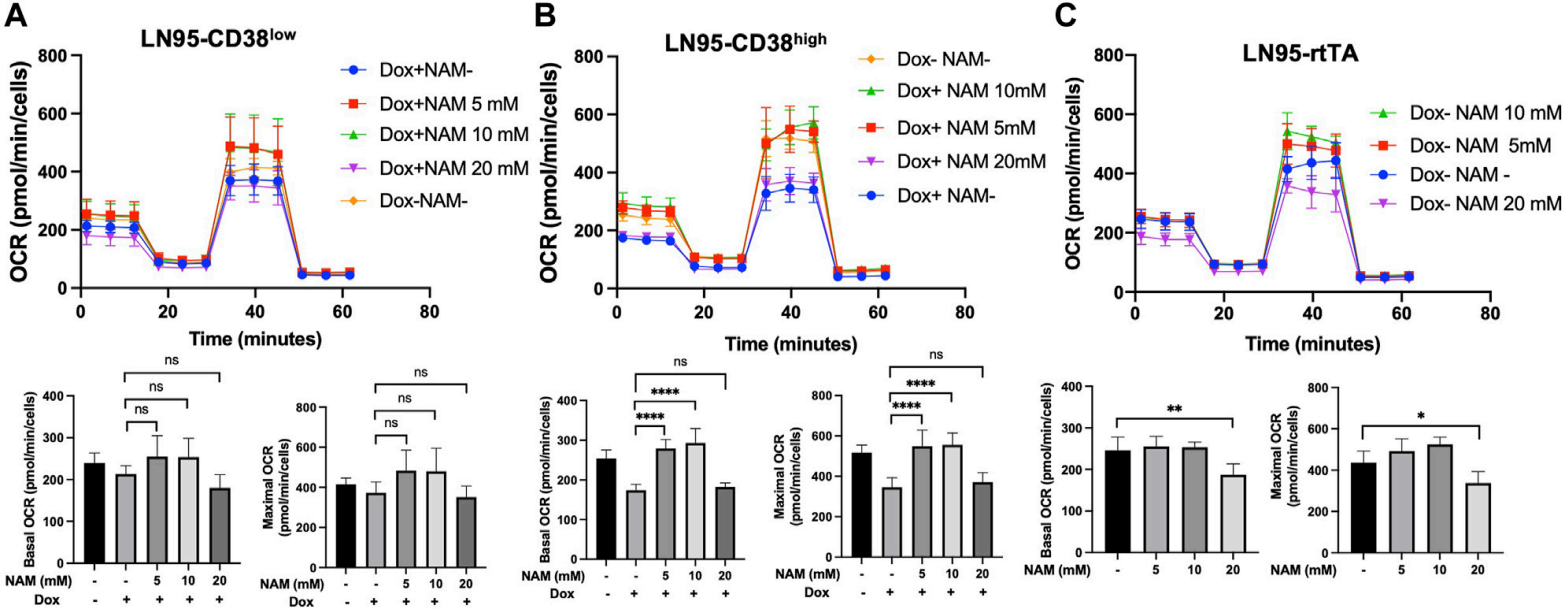
Restoration of mitochondrial respiration by 10 mM NAM. **(A,B,C)** OCR of LN95-CD38^low^ **(A)**, LN95-CD38^high^ **(B)** and LN95-rtTA **(C)** measured by mitochondrial stress test. Basal OCR (measurement point 1) and maximal OCR (measurement point 8) were compared. Mean ± SD values from five replicates are shown. *p* values for Turkey’s multiple comparison tests are indicated in each figure. ns: not significant, *: *p* < 0.05, **: *p* < 0.01,****: *p <* 0.0001.

Given that NAD^+^ is necessary for oxidative phosphorylation in mitochondria and NAM is a NAD^+^ precursor (Chiarugi et al., 2012), we also tested if NAM restores mitochondrial respiration (measured by OCR) that is reduced following CD38 expression. Mitochondrial stress test was performed with or without NAM supplementation. As shown in Figures 5A,B, both LN95-CD38^low^ and LN95-CD38^high^ clones demonstrated a general trend of restored basal and maximal OCR following treatments with 5 and 10 mM NAM, although statistical significance was only observed in the LN95-CD38^high^ clone. Interestingly, 20 mM NAM did not improve OCR, indicating a biphasic dose response to NAM with respect to mitochondrial respiration that may not be linked to apoptosis (see Discussion). We further tested the possible biphasic function of NAM in the Dox-untreated LN95-rtTA clone (i.e., cells not under induced conditions). As shown in Figure 5C, while an increase in mitochondrial respiration as measured by OCR was detected in cells treated with 5 and 10 mM NAM, OCR was significantly decreased in cells treated with 20 mM NAM, in spite of increased intracellular NAD^+^. Taken together, the findings suggest that mitochondrial stress induced by CD38 expression can be restored by NAM, a known NAD + precursor, but only at optimal concentrations (e.g., 5 mM or 10 mM NAM). Higher NAM concentrations (e.g., 20 mM) may affect mitochondrial respiration independently of CD38 expression, apoptosis inhibition and NAD^+^ availability.

## Discussion

In this study, we focused on the functional impact of CD38 overexpression and subsequent NAM supplementation in prostate cancer using a doxycycline-inducible castration-resistant prostate cancer cell line model (LNCaP95). The 2 cell clones expressing different amount of induced CD38 (LN95-CD38^low^ and LN95-CD38^high^), as well as two control clones that do not express CD38, allowed analysis of cellular phenotypes attributable to CD38 expression. The observed CD38-associated phenotypes were largely limited to the clones with CD38 expression following induction and tend to be more prominent in the clone expressing higher level of CD38 (LN95-CD38^high^), confirming that those phenotypes are *bona fide* phenotypes of CD38 expression as opposed to random clone-specific events or artifacts caused by toxic effects of overexpressed exogenous proteins. Using this controlled cell model system, we show that CD38 induces apoptosis, depletes intracellular NAD^+^ and compromises mitochondrial respiration. In addition, we show that nicotinamide (NAM, aka vitamin B3), a known NAD^+^ precursor, can rescue some of the CD38-induced phenotypes as NAM treatments inhibited apoptosis induced by CD38 and restored mitochondrial respiration at optimal concentrations.

Given that NAD^+^-mediated signaling events include transcription, cell cycle progression and metabolic regulation (Chiarugi et al., 2012), silencing CD38, a key molecule that consumes NAD^+^, is seemingly the right strategy for fast-dividing cancer cells to increase NAD^+^ availability (Mottahedeh et al., 2018). This notion is consistent with our finding that NAD^+^ depletion by CD38, in turn, was fatal to prostate cancer cells. Although nicotinamide generated from NAD^+^ breakdown (by CD38) may be recycled for NAD^+^ synthesis through the salvage pathway, the observation that NAD^+^ was depleted following CD38 overexpression suggest that the salvage pathway is not effective in compensating the acute depletion of NAD^+^ by CD38. NAD^+^ depletion can also be achieved by inhibition of key molecules in the salvage pathway. For example, inhibition of nicotinamide phosphoribosyltransferase (NAMPT, a rate-limiting enzyme) by FK866 depletes NAD^+^ and induces apoptosis (Hasmann and Schemainda, 2003). Therefore, CD38 and NAMPT mediate opposite functions with respect to regulation of NAD^+^ levels. Interestingly, while expression of CD38 is lost in advanced prostate cancers, the expression of NAMPT is increased (Chowdhry et al., 2019). Likewise, concurrent overexpression of CD38 and NAMPT inhibition had synergistic effect on growth suppression in pancreatic cancer (Chini et al., 2014), suggesting a critical dependence of cancers on NAD^+^ that may be explored therapeutically.

Our studies attempted to address contradictory findings regarding regulation of intracellular NAD^+^ by CD38 (Chmielewski et al., 2018; Mottahedeh et al., 2018). Determining whether CD38 regulates intracellular NAD^+^ level is functionally important because if confirmed, a cell-autonomous mechanism of CD38 loss, rather than a function mediated through the tumor microenvironment, may be inferred. Mottahedeh et al. (2018) reported that CD38 reduced extracellular NAD^+^ level but did not affect intracellular NAD^+^ level, whereas Chmielewski et al. (2018) showed that CD38 reduced intracellular NAD^+^. Our results are in general in agreement with Chmielewski et al.’s results showing that CD38 reduced intracellular NAD^+^. The discrepancy in their findings can be due to different cell lines/types used for experiments and the amount of CD38 expressed. In Mottahedeh et al.’s study, immortalized benign prostate epithelial cell line RWPE1 and benign prostate tissues derived from CD38 knockout mice were used, whereas prostate cancer cell lines were used for Chmielewski et al.’s study and the present study. It is possible that malignant cells have higher NAD^+^ demands intracellularly, making them more susceptible to CD38-mediated NAD^+^ depletion. Regarding the amount of CD38 overexpressed in cell models, it is difficult to compare the expression level among different studies. In our study, a stable line expressing lower level of CD38 (LN95-CD38^low^) showed more “mild” phenotypes in our study, suggesting that the expression level does affects the functional read-outs.

We found that while NAM suppressed CD38-mediated apoptosis at all concentrations tested, NAM supplementation did not increase intracellular NAD^+^ of CD38-expressing LN95-CD38^low^ and LN95-CD38^high^ cells. Given that NAM did increase NAD^+^ and NAD^+^/NADH ratios to certain extent in CD38 negative cells (i.e., Dox-untreated cells and control LN95-rtTA cells), the finding may be explained by rapid consumption of NAD^+^ synthesized through the salvage pathway due to presence of overexpressed CD38, and it may not be necessary for newly synthesized NAD^+^ to reach high steady levels to inhibit apoptosis and restore mitochondrial respiration.

We also found that while 5 and 10 mM NAM restored mitochondrial respiration impaired by CD38, 20 mM NAM did not improve mitochondrial respiration in Dox-treated LN95-CD38^low^ and LN95-CD38^high^ cells. This observation is consistent with the finding in the control LN95-rtTA not expressing CD38, in which 20 mM NAM suppressed mitochondrial respiration despite an increase of NAD^+^ induced at this concentration. This biphasic dose response phenomenon (aka hormesis) characterized by low dose stimulation and a high dose inhibition is commonly seen in pharmacological agents (Calabrese, 2013). In the case of NAM, NAM exerts both stimulatory and inhibitory effects in regulation of NAD^+^-dependent enzymes (PARPs, CD38 and sirtuins). On the one hand, NAM acts as an NAD^+^ precursor to increase substrate pools (Chiarugi et al., 2012), and on the other hand, NAM (not converted to NAD^+^) inhibits NAD^+^-dependent enzymes by direct binding (Virág and Szabó, 2002; Avalos et al., 2005; Yang and Sauve, 2006). The contradicting roles of NAM beg the question of what determines NAM’s effect on cells. In cell-free system using recombinant proteins, NAM inhibits PARPs and sirtuins in a dose-dependent manner (Rankin et al., 1989; Bitterman et al., 2002). However, in cell-culture conditions, 3-fold higher NAM is required to inhibit PARP1 in comparison with cell-free system (Rankin et al., 1989). Another study using cell lines showed that high dose NAM (≥5 mM) inhibits PARP1, while low dose NAM (<3 mM) enhances DNA repair by increasing intracellular NAD^+^ (Riklis et al., 1990). Together, these findings indicate that NAM is more likely to act as an NAD^+^ precursor at low concentrations but its role leans towards inhibitors of NAD^+^-dependent enzymes at higher concentrations. Therefore, our findings can be explained by some unknown toxic effects of NAM (e.g., excessive suppression of PARPs and sirtuins) that may outweigh its beneficial effects as NAD^+^ precursor at high concentrations. Optimal dosing of NAM may be therapeutically explored to modulate cellular metabolic functions leading to selective anti-cancer activities.

In summary, our studies demonstrated the functional impact of CD38 overexpression and NAM supplementation on prostate cancer cell viability, intracellular NAD^+^ level, and mitochondrial respiration. Additional studies are needed to further elucidate the mechanistic and therapeutic implications of these findings.

## Data Availability

The original contributions presented in the study are included in the article/Supplementary Material, further inquiries can be directed to the corresponding author.
